# Attitudes toward home-based malaria testing in rural and urban Sierra Leone

**DOI:** 10.1186/s12936-015-0582-x

**Published:** 2015-02-15

**Authors:** Shamika Ranasinghe, Rashid Ansumana, Joseph M Lamin, Alfred S Bockarie, Umaru Bangura, Jacob AG Buanie, David A Stenger, Kathryn H Jacobsen

**Affiliations:** Department of Global and Community Health, George Mason University, 4400 University Drive 5B7, Fairfax, VA 22030 USA; Mercy Hospital Research Laboratory, Bo, Sierra Leone; Njala University, Bo, Sierra Leone; US Naval Research Laboratory, Washington, DC 20375 USA; Liverpool School of Tropical Medicine, Pembroke Place, Liverpool, L3 5QA UK

**Keywords:** Malaria, Rapid diagnostic test, Diagnostic test kits, Community health services, Community health worker, Sierra Leone, West Africa

## Abstract

**Background:**

The purpose of this study was to examine malaria testing practices and preferences in Bo, Sierra Leone, and to ascertain interest in and willingness to take a home-based rapid diagnostic test administered by a community health volunteer (CHV) or a trained family member rather than travelling to a clinical facility for laboratory-based testing.

**Methods:**

A population-based, cross-sectional survey of 667 randomly-sampled rural households and 157 urban households was conducted in December 2013 and January 2014.

**Results:**

Among rural residents, 69% preferred a self/family- or CHV-conducted home-based malaria test and 20% preferred a laboratory-based test (with others indicating no preference). Among urban residents, these numbers were 38% and 44%, respectively. If offered a home-based test, 28% of rural residents would prefer a self/family-conducted test and 68% would prefer a CHV-assisted test. For urban residents, these numbers were 21% and 77%. In total, 36% of rural and 63% of urban residents reported usually taking a diagnostic test to confirm suspected malaria. The most common reasons for not seeking malaria testing were the cost of testing, waiting to see if the fever resolved on its own, and not wanting to travel to a clinical facility for a test. In total, 32% of rural and 27% of urban participants were very confident they could perform a malaria test on themselves or a family member without assistance, 50% of rural and 62% of urban participants were very confident they could perform a test after training, and 56% of rural and 33% of urban participants said they would pay more for a home-based test than a laboratory-based test.

**Conclusion:**

Expanding community case management of malaria to include home testing by CHVs and family members may increase the proportion of individuals with febrile illnesses who confirm a positive diagnosis prior to initiating treatment.

## Background

Presumptive treatment of suspected cases of malaria based on symptoms is common in many endemic areas. In Bo, Sierra Leone, more than half of adults who think they have malaria make presumptive self-diagnoses and then treat at home, usually with herbs or with pharmaceuticals purchased without prescriptions in local markets [1]. The 2013 Malaria Indicator Survey in Sierra Leone found that 29% of urban children and 41% of rural children were not taken to a healthcare facility when they had fevers [2]. These statistics indicate a high rate of malaria treatment without testing. Treating suspected malaria without first confirming the presence of *Plasmodium* parasites may lead to a variety of negative individual and community health outcomes due to the misuse of anti-parasitic medications, money wasted on ineffective or unnecessary treatments, and delayed therapy for the actual cause of the fever when the illness is not due to malaria [3]. The World Health Organization and global health partnerships recommend the use of diagnostic testing to avoid unnecessary treatment [4]. Initiatives such as ‘test-treat-track’ promote testing all persons suspected to have malaria, treating with anti-malarial drugs only those who have a positive malaria test result, and tracking progress toward malaria control through health surveillance systems [5].

Two main types of malaria tests are currently available, microscopy and rapid diagnostic tests (RDTs). Microscopic diagnosis is based on visualizing *Plasmodium* parasites in stained and magnified blood smears. Although microscopy is still considered the gold standard for malaria testing because it shows the actual presence or absence of the parasites and allows the parasite load to be quantified [6], microscopes are usually only available at clinical laboratories. Microscopy is also dependent on the skill of the laboratory technician, and the sensitivity and specificity of the test may vary considerably depending on the person performing the examination [6,7].

RDTs do not visualize the parasite but instead detect antigens present in a small sample of blood applied to a test strip [6,7]. Within about five to 20 minutes of applying the blood of a person with malaria to a test strip, one or more lines will appear in the window of the test kit indicating if parasitic antigens have been detected [6,7]. One line usually indicates a negative test result, and two (or more) lines indicate the presence of the antigen [6]. The results may also indicate an invalid result, which would require the test to be re-administered with a new kit. RDTs are a type of point-of-care test (POCT) or point-of-need test [8]. While RDTs are most often used at clinical facilities, they can also be used in home and community settings by community health workers (CHWs) and community health volunteers (CHVs). POCTs are useful when they are cost-effective and accurate tools for providing rapid diagnostic results [8]. Studies in a variety of settings have demonstrated that malaria RDTs have adequately high sensitivity and specificity [6] and can be cost-effective when compared to microscopy [9-12].

Although diagnosis prior to treatment is strongly encouraged by clinical guidelines, there are a variety of factors that limit access to and uptake of malaria testing. Cultural and community beliefs about blood and perceptions of healthcare providers may discourage testing [13], lower-income households may not be able to afford testing or may not prioritize testing when budgeting for healthcare expenses [3,14], and rural residents may delay seeking care from the formal medical system because of the costs and time associated with travelling to a healthcare facility [15,16]. The lack of physical or geographical access to testing is one that can be at least partly addressed by programmes offering community case management (CCM) of malaria. CCM may also overcome some cultural and economic barriers [4,17].

CCM programmes empower CHWs and CHVs to offer basic, low-cost healthcare services to households that might not otherwise have routine access to medical assistance. CHWs and CHVs participating in CCM programmes for malaria perform two key functions: using RDTs to confirm suspected malaria cases and then dispensing anti-malarial drugs to those who test positive for malaria with an RDT [18]. The RDT kits used by CHWs and CHVs generally include disposable gloves, alcohol swabs, a lancet for drawing a small amount of blood, the test kit itself, any buffer solutions necessary for the test, and a small container for safely disposing of the lancet and other hazardous materials [19]. CHWs and CHVs also maintain stocks of commonly needed medications. The ability to use RDTs outside of a laboratory-based setting may increase the number of febrile persons willing to take a malaria test.

The purpose of this study was to use a cross-sectional population-based sampling approach to examine malaria testing practices, attitudes towards malaria testing, and preferences for testing location in rural and urban Bo, Sierra Leone.

## Methods

### Study area

This study was conducted in the city of Bo, Sierra Leone’s second largest urban area, and in surrounding rural areas within the Bo District. Sierra Leone is located on the coast of West Africa, and Bo District is located in the nation’s Southern Province. Falciparum malaria is endemic in Sierra Leone, and the country has one of the highest prevalence rates of malaria in the world [4]. Malaria diagnosis (usually through microscopy rather than RDTs) and artemisinin-based combination therapy (ACT) are available free to users of all ages from public providers [4]. There are few healthcare facilities in rural areas, but the city of Bo is home to a diversity of public and private outpatient clinics and several hospitals offering both inpatient and outpatient services [20,21].

### Study population

In 2009, Mercy Hospital Research Laboratory (MHRL) used a participatory geographic information system (GIS) process to map the entire city of Bo, including the locations of all residential, public and commercial structures [22]. The GIS has been regularly updated since then. This database enabled MHRL to use a geography-based sampling method to recruit households from Bo city for this study. A two-stage cluster (neighbourhood-based) sampling method was used to identify urban participants. First, seven of 68 sections (neighbourhoods) in the city of Bo were randomly sampled, and then 30 households from each of those seven sections were randomly sampled for participation. In the rural areas, 23 rural communities (villages) were randomly sampled for participation from a list of the 1,266 villages in Bo District. Interviewers visited these communities to recruit participants. When fewer than 30 households were located within the community, interviewers visited residences at the periphery of the villages until they had contacted 30 households. In total, 824 of the 900 (91.6%) contacted households participated in the study, including 667 of 690 (96.7%) rural households and 157 of 210 (74.8%) urban households.

In each of the 824 participating households, one adult representative (age 18 or older) was asked to participate in a study of attitudes and practices related to malaria testing. The interviewers preferentially recruited household members who said they were febrile at the time of data collection. In both the urban and rural areas, the interviewers alternated between asking to interview a female household member and requesting an interview with a male household member. If an adult of the preferred gender was not available, a household member of the opposite gender was interviewed instead. Responses to interviewer questions were recorded in an OpenDataKit questionnaire installed on smartphones. All interviews were completed between 14 December, 2013 and 31 January, 2014.

### Questionnaire

Each participant was asked whether he or she was currently febrile or without fever. Based on this response, the next questions related to either the current fever or to the most recent febrile illness the participant had experienced. The first set of questions asked whether a diagnostic test had been performed to determine the cause of the fever. Participants who did not complete a diagnostic test during the current or most recent febrile illness were asked a series of yes/no questions about their reasons for not seeking a microbiologically-confirmed diagnosis. The next set of questions asked whether the participant consulted a doctor or other healthcare professional about the fever and, if so, asked about the clinician’s recommendations for testing. Participants were asked about their attitudes about home-based self-testing, CHV-assisted home-based testing and laboratory-based malaria testing. Data were also collected about demographics, socio-economic status and self-rated quality of life.

### Ethical considerations

The study was approved by the institutional review boards of Njala University, George Mason University, the Liverpool School of Tropical Medicine, the US Naval Research Laboratory, and the Office of the Sierra Leone Ethics and Scientific Review Committee (SLESRC). Participation was voluntary, and no incentives or compensation for participation were provided. All participants provided a thumbprint as documentation of their informed consent.

### Analysis

The primary variable of interest for this analysis was a preference for home-based testing, whether conducted by self/family or by a CHV, over laboratory-based testing for suspected cases of malaria. Participants who answered both the “self/family vs. laboratory” and “CHV vs. laboratory” questions with a preference for home-based testing were considered to have a strong preference for home-based testing, and participants who answered both of these questions with a preference for laboratory-based testing were considered to have a strong preference for laboratory-based testing. Participants indicating no preference, those who would not take the test at either venue, and those who preferred home-based testing for one of the paired-comparison questions and laboratory-based testing for the other were not considered to have a strong preference for home-based or laboratory-based testing and were excluded from the analyses that focused on only participants with consistent preference for one of the two testing settings. Participant characteristics and testing preferences were compared using two-sided Pearson’s Chi-squared tests. Because there were significant differences in the characteristics of rural and urban participants, most analyses were conducted separately by location. The statistical analysis was conducted using SPSS version 22 with a significance level of α = 0.05.

## Results

The age and gender distributions of rural and urban participants were similar (Table 1). The mean age of the participants was 40.9 years (standard deviation: 18.3), with half of the participants male and half female, as per the study design. However, there were significant socio-economic differences by location, with rural residents reporting lower levels of education (p < 0.001), lower rates of literacy (p < 0.001) and less ownership of mobile phones (p < 0.001). Rural residents were also more likely than urban residents to rate their quality of life and their health status as poor (p < 0.01). Because of these significant differences by location, all analyses were conducted separately for rural and urban participants.

**Table 1:** **Characteristics of participants in rural and urban areas (n = 824)**

**Characteristic**		**Rural (n = 667)**		**Urban (n = 157)**		**2-sided Chi-squared test ** **p-value**
		**n**	**%**	**n**	**%**	
Gender	Male	335/667	50.2	77/157	49.0	0.790
	Female	332/667	49.8	80/157	51.0	
Age (years)	18-29	178/650	27.4	57/157	36.3	0.087
	30-49	276/650	42.5	59/157	37.6	
	≥50	196/650	30.2	41/157	26.1	
Years of formal education	No formal school	436/665	65.6	93/155	60.0	**<0.001**
	1-7 years	109/665	16.4	8/155	5.2	
	8-11 years	62/665	9.3	23/155	14.8	
	≥12 years	58/665	8.7	31/155	20.0	
Can read English		172/650	26.5	61/155	39.4	**0.001**
Can read Arabic		44/655	6.7	24/154	15.6	**<0.001**
Comfortable reading a newspaper		123/473	26.0	54/116	46.6	**<0.001**
Owns a mobile phone		208/661	31.5	77/152	50.7	**<0.001**
Actively involved in a faith-based organization		601/657	91.5	135/145	93.1	0.519
A HCP in Bo is a family member or friend		287/664	43.2	84/154	54.5	**0.011**
Quality of life over the past four weeks	Good or very good	213/666	32.0	50/157	31.8	**0.007**
	Average	241/666	36.2	75/157	47.8	
	Poor or very poor	212/666	31.8	32/157	20.4	
Satisfaction with health over the past four weeks	Satisfied or very satisfied	303/667	45.4	86/157	54.8	**0.001**
	Average	162/667	24.3	46/157	29.3	
	Dissatisfied or very dissatisfied	202/667	30.3	25/157	15.9	
Comparison of health status to the average person of the same age	More healthy	181/667	27.1	56/157	35.7	0.097
	About the same	201/667	30.1	44/157	28.0	
	Less healthy	285/667	42.7	57/157	36.3	

HCP: healthcare provider.

The numbers in bold are statistically significant test results (p<0.05).

Participant answers to the paired-comparison questions about preferred malaria testing location expressed a significant preference for home-based testing over laboratory-based testing in rural areas (Table 2). Among the 660 rural residents who responded to these questions, 68.8% (n = 454) preferred home-based testing by self/family or by a CHV over laboratory-based testing; 19.8% (131) preferred laboratory-based testing over home-based testing; 4.2% (28) rated laboratory-based testing as better than CHV-assisted testing but worse than self/family testing; 2.6% (17) rated laboratory-based testing as better than self/family testing but worse than CHV-assisted testing; and 4.5% (30) indicated no preference or said they would decline all of these testing options. Among the 154 urban residents who responded, 38.3% (59) preferred home-based testing over laboratory-based testing and 43.5% (67) preferred laboratory-based testing over home-based testing. In both rural and urban areas, CHV-assisted home-based testing was preferred over self/family-conducted home-based testing. In rural areas, 68.1% (450) preferred CHV-assisted testing compared to only 28.1% (186) who preferred self/family-conducted testing. In urban areas, 76.8% (119) preferred testing by a CHV and 21.3% (33) preferred testing by self or a family member.

**Table 2:** **Preferences for malaria test location (n = 824)**

**Question**		**Home-based test by self/family**	**Home-based test by a trained CHV**	**Laboratory-based test**	**No preference**	**Would not take the test either way**
Some communities have trained volunteers who are able to do malaria tests in their neighbours’ homes. If a community health volunteer lived in your section, would you prefer to take a malaria test at a clinical laboratory or in your home?	Rural	--	72.1% (477/662)	24.8% (164/662)	1.7% (11/662)	1.5% (10/662)
	Urban	--	40.9% (63/154)	58.4% (90/154)	0.6% (1/154)	0.0% (0/154)
Some new malaria tests are designed for self-use. If you had the option of testing yourself for malaria at home, would you prefer to take a malaria test at a clinical laboratory or in your home?	Rural	73.5% (486/661)	--	22.5% (149/661)	2.0% (13/661)	2.0% (13/661)
	Urban	52.6% (81/154)	--	46.1% (71/154)	1.3% (2/154)	0.0% (0/154)
If you were going to take a malaria test while at home, would you prefer to do the test yourself (or have a family member help you) or have a community health volunteer assist you?	Rural	28.1% (186/661)	68.1% (450/661)	--	2.0% (13/661)	1.8% (12/661)
	Urban	21.3% (33/155)	76.8% (119/155)	--	0.6% (1/155)	1.3% (2/155)

Note: This table excludes participants who did not provide a response to either question.

In total, 47.8% (n = 308/644) of the rural participants and 75.3% (116/154) of the urban participants said they usually consult with a healthcare provider about suspected malaria (Table 3). Among rural and urban participants, 37.1% (247/666) and 55.8% (87/156), respectively, reported having had a diagnostic test to determine the cause of the most recent febrile illness, 35.6% (231/649) and 62.7% (94/150) reported usually taking a diagnostic test to confirm suspected malaria, and 89.5% (578/646) and 88.2% (125/153) said that they usually bought medications or herbs to treat malaria without first consulting with a healthcare provider.

**Table 3:** **Characteristics of participants who indicated a consistent preference for home-based testing (by self/family or CHV) or laboratory-based testing**

**Characteristic**		**Rural**			**Urban**		
		**Home-based testing preference (n = 454)**	**Laboratory-based testing preference (n = 131)**	**2-sided Chi-squared test** **p-value**	**Home-based testing preference (n = 59)**	**Laboratory-based testing preference (n = 67)**	**2-sided Chi-squared test** **p-value**
Febrile at the time of the survey		52.4 (238/454)	55.7 (73/131)	0.505	39.0 (23/59)	40.3 (27/67)	0.880
Usually consults with a healthcare provider about suspected malaria		45.5 (201/442)	56.0 (70/125)	**0.038**	71.2 (42/59)	78.8 (52/66)	0.326
A healthcare provider recommended a diagnostic test during the most recent consultation for a fever		55.2 (232/420)	54.2 (64/118)	0.847	62.7 (37/59)	80.0 (52/65)	**0.033**
Had a diagnostic test to determine the cause of most recent febrile illness		34.0 (154/454)	45.8 (60/131)	**0.014**	47.5 (28/59)	63.6 (42/66)	0.069
Usually takes a diagnostic test to confirm suspected malaria		32.5 (145/446)	43.2 (54/125)	**0.027**	39.7 (23/58)	77.8 (49/63)	**<0.001**
Usually buys medicines to treat malaria without first consulting a healthcare provider		82.7 (369/446)	64.3 (83/129)	**<0.001**	89.8 (53/59)	82.1 (55/67)	0.215
Willing to pay more for a home-based test than a laboratory-based test		63.2 (287/454)	25.4 (33/130)	**<0.001**	44.1 (26/59)	20.9 (14/67)	**0.005**
Belief about how often adult fevers in the community are due to malaria	Always	30.2 (137/454)	11.5 (15/131)	**<0.001**	35.6 (21/59)	20.9 (14/67)	0.160
	Most of the time	27.3 (124/454)	62.6 (82/161)		39.0 (23/59)	52.2 (35/67)	
	Sometimes	35.5 (161/454)	20.6 (27/131)		25.4 (15/59)	26.9 (18/67)	
	Rarely or never	7.0 (32/454)	5.3 (7/131)		0.0 (0/0)	0.0 (0/0)	
Confidence in ability to perform a malaria test on self without assistance	Very confident	36.5 (165/452)	26.1 (29/111)	**0.034**	45.8 (27/59)	16.4 (11/67)	**<0.001**
	Somewhat confident	17.7 (80/452)	14.4 (16/111)		45.8 (27/59)	26.9 (18/67)	
	Not at all confident	45.8 (207/452)	59.5 (66/111)		8.5 (5/59)	56.7 (38/67)	
Confidence in ability to perform a malaria test if trained on how to use a test kit	Very confident	51.9 (235/453)	43.2 (48/111)	**0.005**	83.1 (49/59)	37.3 (25/67)	**<0.001**
	Somewhat confident	15.5 (70/453)	28.8 (32/111)		13.6 (8/59)	23.9 (16/67)	
	Not at all confident	32.7 (148/453)	27.9 (31/111)		3.4 (2/59)	38.8 (26/67)	

The numbers in bold are statistically significant test results (p<0.05).

Rates of preference for home-based testing and for laboratory-based testing were similar in rural and urban areas by gender (p = 0.488 and 0.713, respectively), age (p = 0.670 and 0.605), and years of education (p = 0.094 and 0.039, with urban residents with more education more likely to prefer laboratory-based testing). There was no difference in testing location preference by fever status at the time of the survey (p = 0.929). Among rural residents, a preference for home-based testing over laboratory-testing was especially high among those who were not comfortable reading a newspaper (p = 0.006), those not owning a mobile phone (p = 0.031), those who reported usually self-treating suspected malaria rather than consulting with a healthcare provider (p < 0.001), and those who believe that adult fevers are always caused by malaria (p < 0.001). Among urban residents, a preference for home-based testing over laboratory-testing was especially high among those who were able to read English (p = 0.003) and comfortable reading a newspaper (p = 0.001), those who did not usually take a diagnostic test to confirm suspected malaria (p < 0.001), and those who reported being very confident about their ability to perform a malaria self-test after training (p < 0.001). Those who indicated a preference for home-based testing were significantly more likely than those preferring laboratory-based testing to say that they would be willing to pay extra for the option of a home-based test, and this was found in both rural (p < 0.001) and urban (p = 0.005) areas.

In total, 31.7% (198/625) of rural participants and 27.3% (42/154) of urban participants said they were very confident they could perform a malaria test on themselves or a family member without assistance. Among those indicating a preference for self/family-conducted home-based testing over CHV-assisted testing, 74.2% (138/186) of rural residents and 33.3% (11/33) of urban residents were very confident in their ability to conduct a test on themselves or a family member. Reported self-confidence was lower among participants indicating a preference for a home-based tested administered by a CHV rather than self/family, with only 14.0% (60/428) of rural residents and 26.1% (31/119) or urban residents with this preference saying they were very confident in their self-testing ability. In total, 50.1% (314/627) of the rural participants were very confident they could perform a test if they had been trained on how to use a test kit, including 88.7% (165/186) with a preference for self/family-conducted home-based testing and 34.0% (146/430) with a preference for testing administered by a CHV. Among the urban participants, 61.7% (95/154) were very confident in their testing abilities if trained, including 75.8% (25/33) with a preference for self/family-conducted home-based testing and 58.8% (70/119) with a preference for testing administered by a CHV.

Among the 488 participants who did not seek a diagnostic test for their most recent febrile illness, 330 (67.6%) preferred a home-based test and 95 (19.5%) preferred a laboratory-based test (Table 4). The most common reasons for not seeking testing were similar for both testing preference and location groups: not wanting to spend money on the test (68.0%), waiting to see if the fever resolved within a few days (45.0%), not wanting to travel to a clinical facility for a test (41.0%) and assuming that a formal diagnosis was not needed because adults could presumptively diagnose the cause of the fever (37.4%). Those with a preference for home-based testing were more likely than those with a preference for laboratory-based testing to say that they did not need a test because they already knew the cause of the fever (p = 0.025). Urban non-testers were significantly more likely than rural non-testers to assume that they already knew the cause of the fever (p = <0.001) and to say that they did not want to wait for test results (p = 0.010). A dislike of blood draws was not a common reason not to seek testing (15.6%).

**Table 4:** **Reasons for not seeking diagnostic testing for the last febrile illness**

**Characteristic**	**Home-based (by self/family or CHV) testing preference (n = 330)**		**Laboratory-based testing preference (n = 95)**	
	**Rural (n = 299)**	**Urban (n = 31)**	**Rural (n = 71)**	**Urban (n = 24)**
Did not want to spend money on the test	64.4 (192/298)	71.0 (22/31)	72.9 (51/70)	82.6 (19/23)
Wanted to wait a few days to see if the fever went away	42.4 (126/297)	63.3 (19/30)	32.8 (22/67)	52.4 (11/21)
Did not want to travel to a laboratory	41.9 (124/296)	54.8 (17/31)	22.5 (16/71)	60.9 (14/23)
Did not need a test because already knew the cause of the fever	34.5 (102/296)	83.9 (26/31)	18.6 (13/70)	52.4 (11/21)
A doctor or other healthcare provider did not recommend a test	27.2 (81/298)	25.8 (8/31)	33.3 (23/69)	31.6 (6/19)
Did not want to have to wait for the test results	23.5 (70/298)	35.5 (11/31)	16.9 (12/71)	56.5 (13/23)
Does not like blood draws	14.2 (42/296)	16.1 (5/31)	11.4 (8/70)	34.8 (8/23)

Note: This table presents results for the 488 (59.4%) adults who reported not seeking microbiological diagnosis of the cause of the most recent febrile illness and then indicated a preference for home-based or laboratory-based testing (n = 425).

## Discussion

The participants in this study, especially those living in rural areas, expressed a high level of interest in home-based testing for malaria. A recent systematic review of 27 studies of CCM of malaria found consistent evidence that trained CHWs could successfully conduct RDTs and use the test results to recommend appropriate treatment [18]. Studies in which community members are part of the selection process for CHVs are particularly successful in increasing access to testing [10,17]. The primary concerns about CCM of malaria are some CHWs recommending anti-malarial medications following negative malaria tests and many febrile individuals not following through on recommended clinical consultations after negative test results [18,23]. Few studies have examined the use of home-based RDTs conducted by family members trained and supervised by CHVs rather than by the CHVs themselves, but this study suggested an openness to self/family testing in addition to CHV testing. Self/family testing with RDTs pre-purchased by the household might increase malaria-testing access in small farming villages that are located farther than easy walking distance from a community with a trained CHV.

Home-based malaria testing, often with the assistance of a CHV, has been successfully implemented in other sub-Saharan African communities. In the rural Iganga district of Uganda, community members expressed a high level of trust in CHVs who were trained to conduct malaria RDTs and to recommend appropriate malaria treatment [24]. In villages in the South Kordofan State of Sudan, the use of CHV-conducted RDTs significantly increased access to malaria tests and malaria treatment, especially during the rainy season when travel to a clinic was often impossible [17]. In two provinces in Zambia, CHVs had a very high rate of correct recommendations for malaria treatment or referral to a healthcare facility after conducting in-home RDTs [10]. Given the substantial interest in home-based malaria testing in rural Bo district in Sierra Leone, it seems likely that CHVs in these communities could successfully test and treat malaria in homes, and perhaps even train willing and confident family members to conduct RDTs in their own households, making appropriate referrals for hospital care when necessary.

The most commonly reported barriers to the uptake of home-based testing for malaria include concerns about infection transmission during blood draws [24,25] and concerns about reliable access to RDTs for home-based testing use [10,23,26]. Any programme for home-based testing would need to include clear instructions about how to safely dispose of lancets and test kits. CHVs could be encouraged to collect used kits they had distributed and to ensure that they were processed as medical waste. Consistent access to RDTs would need to be supported through community partnerships with local hospitals and government health officials.

This study in Sierra Leone used a cross-sectional approach with self-reported answers about febrile status and testing preferences. Participants were not asked to try conducting a malaria test on themselves or a family member, so all answers about willingness to conduct a test and confidence about the ability to conduct a test are hypothetical. This willingness-to-test survey will need to be followed by field tests of RDTs by CHVs and CHV-trained family members. Should these tests prove to be acceptable to the community, safe and valid, the programme could be expanded to include RDTs for other locally-important infections.

## Conclusions

Expanded access to CCM might improve access to diagnosis and appropriate, timely and cost-efficient treatment. Existing CHV networks in Sierra Leone could provide a foundation for intensifying CCM. The success of home-based testing in these communities would likely be dependent on (1) a high level of respect for and trust in new and existing CHVs by the communities they support; (2) the availability of a strong network of CHVs who are confident in their own RDT skills and are able to conduct tests themselves as well as teach others to conduct tests; (3) sufficient public health resources to support ‘train-the-trainer’ sessions to teach CHVs how to coach their neighbours through their first self/family malaria tests; and, (4) a competitive price point for home test kits when compared to the out-of-pocket costs of laboratory-based testing.

The possible importance of home-based self-testing has been highlighted by the recent Ebola outbreak. In the months after this survey was conducted, hundreds of cases of Ebola occurred in Bo district [27,28]. Many clinics and hospitals in Bo closed their doors rather than risk exposing their staff to the Ebola virus, and others were forced to temporarily close because of quarantines. Laboratory-based testing for febrile illnesses became more difficult to conduct and more costly due to the need to assume that all biological specimens could be from a patient with Ebola, even when the most likely diagnosis was something more common and much less fatal. Quarantines meant that some CHVs in rural areas could not travel from their home villages to neighbouring ones or stopped volunteering because they feared contracting Ebola. Thus, access to routine medical care, including diagnosis and treatment of common infections such as malaria, decreased significantly, especially in rural areas that already had limited access to health services. Home-based testing that allows trained individuals with suspected malaria to test themselves and family members at home, thus limiting the exposure of others to their blood, could improve the home-based care that has become necessary for many households as a result of the strain Ebola put on the entire healthcare system of Sierra Leone.

## Abbreviations

CCM: Community case management
CHV: Community health volunteer
CHW: Community health worker
GIS: Geographic information system
HCP: Healthcare provider
MHRL: Mercy Hospital Research Laboratory
POCT: Point-of-care test
RDT: Rapid diagnostic test
